# Impact of Soil Chemical Attributes on the Behavior and Spread of Fusarium Oxysporum in Date Palm

**DOI:** 10.1002/pei3.70095

**Published:** 2025-10-23

**Authors:** Laala Djekiref, Khaled Boukehil, Christian Cilas, Mohammed Tirichine

**Affiliations:** ^1^ Department of Agronomic Sciences, Laboratory of Promotion Innovation of Agriculture in Arid Regions Mohamed Khider University Biskra Algeria; ^2^ AGAP Institut Univ Montpellier, CIRAD, INRAE, Institut Agro Montpellier France; ^3^ Regional Plant Protection Station Ghardaia Algeria

**Keywords:** Bayoud, date palm, epidemic, *Fusarium oxysporum* f. sp. *albedinis*, inoculum dynamics, plant nutrition, soil chemical analysis

## Abstract

Fusarium wilt of date palm ( *Phoenix dactylifera* L.), caused by *Fusarium oxysporum* f. sp. *albedinis*, continues to threaten oases across the Maghreb. However, the disease has failed to establish in Southeastern Algeria, despite the ongoing movement of potentially contaminated plant material. This study investigated whether soil chemical properties contribute to this apparent epidemiological boundary. A total of 48 soil samples were collected from healthy and diseased date palm groves across infected (Adrar, Ghardaïa) and uninfected (Biskra) regions. Soils were analyzed for pH, electrical conductivity (EC), organic matter, calcium carbonate (CaCO_3_), soluble cations (Ca^2+^, Mg^2+^, Na^+^, K^+^), and available phosphorus (PO_4_^3−^). While no consistent differences were observed between healthy and diseased trees within infected areas, five parameters—EC, Ca^2+^, PO_4_^3−^, Mg^2+^, and CaCO_3_—differentiated uninfected from infected regions. Higher levels of EC, Ca^2+^, and CaCO_3_ in uninfected soils suggest a suppressive effect on the pathogen or enhanced host resistance. These findings align with previous studies showing that elevated salinity and calcium can limit Fusarium development by altering cell wall integrity, enzyme activity, and spore production. Phosphorus and magnesium may further modulate disease expression through effects on plant immunity and pathogen metabolism. Our results support the hypothesis that *F. oxysporum* f. sp. *albedinis* is constrained by edaphic factors in Southeastern Algeria. This study highlights the importance of soil chemistry in shaping pathogen distributions and suggests that nutrient‐based management may help suppress Fusarium wilt in date palm agroecosystems.

## Graphical abstract

Mechanisms by which soil elements affect disease expression.

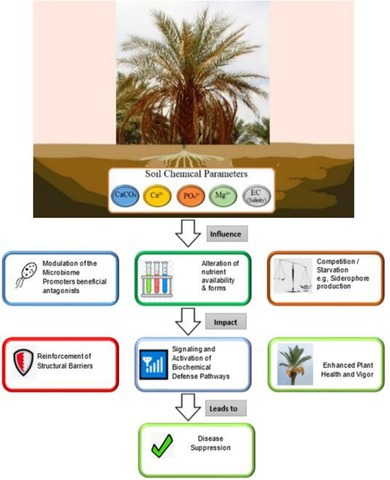

## 1 Introduction

Vascular fusarium wilt of date palms ( *Phoenix dactylifera* L.) is a disease caused by *Fusarium oxysporum* f. sp. *albedinis* (Killian and Maire). This telluric fungus attacks almost all varieties. This disease continues to decimate 4.5%–12% of the world's palm groves annually, causing significant genetic erosion and major production losses (Djerbi 1982, 1988; Sedra 2011). While this disease is a scourge for a large part of North Africa's palm groves, it also represents a permanent epidemiological threat, in human, social, and economic terms, to palm‐growing areas that are free of it. It is therefore essential to identify the factors that promote or hinder the spread of the disease. Ghardaïa has been at the forefront of the disease spread to the eastern regions of the country since it was first reported there in 1965 (Dubost and Kada 1975; Djerbi 1982). The slow progression of the disease over the past 50 years suggests that edaphic factors may limit its spread. The study aims to understand why the disease has not spread to palm groves in southeastern Algeria, despite the absence of strict control of plant material. Among the various environmental parameters that interact with the host plant and the causative agent of the disease, we focused on the soil. The hypothesis that the geographical spread of the disease and the sensitivity of the pathogen, considered an “intruder” in healthy areas, are curbed by edaphic factors can be put forward. This hypothesis is supported by studies showing the influence of soil and its sensitivity on the distribution and dynamics of the inoculum (Stotzky and Pramer 1972; Mitchell 1979; Alabouvette 1983; Alabouvette et al. 1984; Amir and Amir 1988; Campbell 1994). Therefore, understanding the elements that influence the establishment and development of this inoculum is helpful. Furthermore, while disease resistance is genetically regulated, some plant resistance genes are only activated by particular environmental cues. One such stimulus that can have considerable impacts is mineral nutrition (Schumann et al. 2017). Mineral imbalances can determine the resistance or susceptibility of plants to disease, their structure or histological and morphological properties, and the ability of pathogens to survive on and spread within the host (Datnoff et al. 1991; Graham and Webb 1991; Huber 1994; Huber et al. 2012; Guerra et al. 2013; Elmer and Datnoff 2014; Gupta et al. 2017; Carof et al. 2018; Sester et al. 2019). In this context, the impact of the microbiome cannot be overstated. Numerous studies have demonstrated that microbial communities associated with their hosts promote plant growth, enhance nutrient absorption, and bolster resistance to pathogens (Richardson and Simpson 2011; Ramadan et al. 2016; Gouda et al. 2018; Moutassem et al. 2018; Trivedi et al. 2020; Liu et al. 2024). A comparative study is proposed to analyze the differences between soils in healthy and contaminated areas. A presence–absence approach will be applied at three scales:

– Healthy and diseased areas within the same palm grove.
– Healthy and diseased areas between different affected regions.
– Healthy areas in unaffected regions and healthy/diseased areas in affected regions.

It will be possible to isolate the discriminating edaphic factors through the comparison.

The objectives of this work are as follows:

– To determine the impact of soil nutrients on the dynamics and distribution of *F. o. albedinis*.
– To assess the epidemiological risks associated with these soil‐related factors.
– To explore the role of mineral nutrition in the activation of resistance genes in plants, which could explain susceptibility or resistance to the disease.

## 2 Materials and Methods

### 2.1 Study Area and Sampling Design

**FIGURE 1:**
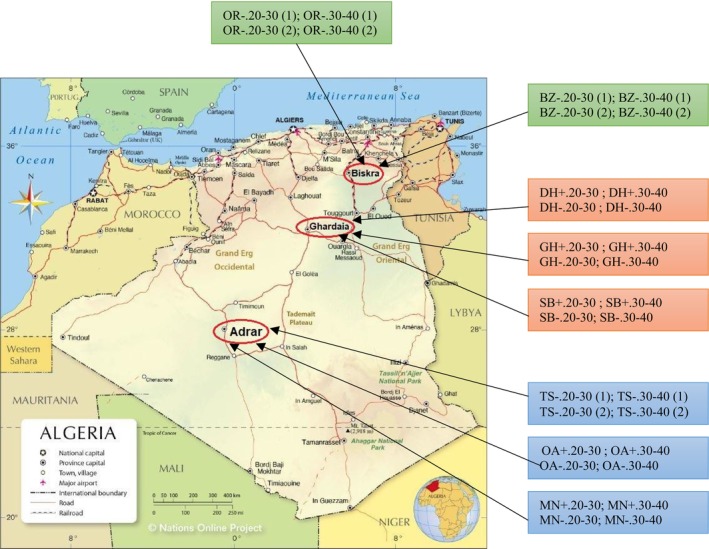
A map indicating the different sample collection sites across the three study areas. BB, commune of Bordj Ben Azzouz; DH, commune of Dahaïa; GH, commune of Ghardaïa; MS, commune Mansouriah‐Timmi; OA, commune Ouled Aïssa‐Timmi; OR, commune of Ourelal; SB, commune of Sebseb; TS, commune T'sabit.

For soil sampling, a random approach was employed across eight palm groves. Six of these groves were infested with Bayoud disease, with three of them located in the Wilaya of Adrar (communes of Ouled Aïssa‐Timmi “OA,” Mansouriah‐Timmi “MN,” and T'sabit “TS”) and the other three in the Wilaya of Ghardaïa (communes of Sebseb “SB,” Dahaïa “DH,” and Ghardaïa “GH”). The remaining two uninfected groves were situated in an intact zone within the Wilaya of Biskra (communes of Ourelal “OR” and Bordj Ben Azzouz “BB”) (Figure 1).

From each infected palm grove, three diseased palm trees and three healthy palm trees (showing no disease symptoms) were selected. In the healthy zone, only three healthy palm trees were sampled. This resulted in a total of 36 palm trees sampled in the infected area (18 healthy and 18 diseased) and six healthy palm trees in the uninfected area.

### 2.2 Soil Sample Collection and Preparation

Soil samples were collected at a distance of 1 m from the stipe of each palm tree at three distinct levels: 0–20 cm, 20–30 cm, and 30–40 cm. The three soil samples obtained from the same horizon of each palm tree within the same palm grove were subsequently blended.

All soil samples were air‐dried for 5 days, with daily stirring. Following drying, they were cleansed, cleared of coarse elements, and sieved to 2 mm. Three aliquots were prepared from each mixture (totaling 126 aliquots) for chemical analysis. These analyses aimed to evaluate, according to established standards, parameters such as pH, electrical conductivity (EC), total limestone, organic matter, soluble cations (Ca^2+^, Mg^2+^, Na^+^, and K^+^), and the concentration of assimilable phosphorus PO_4_^2−^.

### 2.3 Chemical Analyses

The applied chemical analysis methods were based on standard soil analysis laboratory manuals (Pauwels et al. 1992).

#### 2.3.1 pH and Electrical Conductivity (EC)

Soil pH and electrical conductivity were measured electrometrically using a pH meter and a conductivity meter, respectively, with a soil/water ratio of 1/2.5.

#### 2.3.2 Organic Matter (OM)

Organic matter was measured using a spectrophotometer (DR 2000). The method relies on the principle of oxidizing organic matter with an excess quantity of potassium dichromate in a sulfuric medium.

#### 2.3.3 Total Limestone

Total limestone was determined using volumetric calcimetry with a Bernard calcimeter. This method utilizes the property of calcium carbonate CaCO_3_ to disintegrate under the action of an acid, producing carbon dioxide (CO_2_) which is collected in a milliliter‐sized tube. The reaction is represented as:

$${\text{CaCO}}_{3}+2\;\mathrm{HCl}\to {\mathrm{CO}}_{2}+H_{2}O+{\text{CaCl}}_{2}\;\left(\text{Calcium chloride}\right)$$

#### 2.3.4 Dosage of Soluble Cations

The dosage of soluble cations was carried out using a 1/5 aqueous extract.

– Ions Ca^2+^ and Mg^2+^ were measured by a Perkin Elmer atomic absorption spectrophotometer.
– Ions Na^+^ and K^+^ were measured by an Eppendorf flame spectrophotometer.

#### 2.3.5 Assimilable Phosphorus PO_4_^2−^

The Olsen method was applied for assimilable phosphorus dosage. Measurement was performed using spectrophotometry with molybdenum blue. This method is considered the most applicable for estimating assimilable phosphorus in acidic, neutral, and alkaline soils.

The analysis of the results and their interpretation, considering the different factors, was conducted according to standards established in the references (Aubert 1978; Baize 2000; Sarkar and Haldar 2005).

### 2.4 Data Analysis

To highlight any probable variability in chemical composition between healthy and diseased horizons, analyses of variance were performed. These analyses considered nine identified parameters: Organic Matter (M.O), Electrical Conductivity (CE), pH, assimilable phosphorus (PO_4_^2−^), total limestone (CaCO_3_), sodium (Na^+^), potassium (K^+^), calcium (Ca^2+^), and magnesium (Mg^2+^). Boxplots were generated to visualize the observed differences. All analyses were carried out using SAS software (Statistical Analysis System) (SAS Institute Inc 2019).

## 3 Results

According to the raw results of the analyses, the general properties of the different soil samples, classified by zone and by state of disease presence or absence, can be described as follows:

- Infected samples from an infected palm grove in an infected area

For SB+.20–30 and SB+.30–40, these soils were not very salty. The pH ranged from basic (pH 8.27) to slightly alkaline (pH 7.79). These soils were relatively calcareous with low levels of organic matter. The soil solution was dominated by Mg^2+^ and, to a lesser extent, by Na^+^ and Ca^2+^. The K^+^ and PO_4_^2−^ rates indicated low soil fertility.

For DH+.20–30 and DH+.30–40, we noted a low salinity with an EC of less than 0.5 dS/m. The pH was alkaline. The ground was relatively rich in CaCO_3_, but had low organic matter content (< 2%). The soil solution was weakly mineralized, with Mg^2+^ being the most prevalent cation.

The results revealed that the GH+.20–30 and GH+.30–40 soils were weakly saline and basic, respectively. These soils were more or less rich in CaCO_3_. Regarding chemical fertility, the soil had a low level of K^+^. This could be explained by the low levels of organic matter, which resulted in low or limited microbial activity.

The OA+.20–30 and OA+.30–40 soils were not salty, and the pH was slightly alkaline. They were limestone soils with contents greater than 15%. These soils were moderately fertile. The organic matter contents ranged from 1.03% to 14.4%. Soil solutions contained Ca^2+^ as the dominant cation relative to the two horizons that were reached. The K^+^ and the PO_4_^2−^ were below the soil chemical fertility threshold.

Regarding the MN+.20–30 and MN+.30–40, the latter was unsalted with an alkaline pH. They were relatively calcareous with CaCO_3_ content below 15%. The soil was poor chemically and biologically. The soil solution was weakly mineralized with calcium and sodium facies, and secondarily, magnesium facies.

- Healthy samples were taken from an infected palm grove in an infected area

The two horizons, SB−.20–30 and SB−.30–40, expressed a low salinity, ranging from 0.14 to 0.29 dS/m. The pH ranged from slightly alkaline to alkaline. The organic matter content, including PO_4_^2−^ and K^+^, was around the fertility threshold. The geochemical facies were marked by the presence of Ca^2+^ and Mg^2+^.

DH−.20–30 and DH−.30–40 were weakly salty soils. The pH was alkaline. We also noted low biological fertility, with organic matter content ranging from 0.32% to 0.39%. The solution had low concentrations of PO_4_^2−^ and K+, while Mg^2+^ and Ca^2+^ were present in relatively average concentrations.

GH−.20–30 and GH−.30–40: These soils were slightly salty. The pH was slightly alkaline to alkaline. A CaCO_3_ content of > 15% revealed a carbonate state in the soil. The level of organic matter was low. The K^+^ and PO_4_^2−^ contents were also low. The solution's chemical properties were sodium and calcium. We therefore suggest that high pH levels depend on a high concentration of HCO_3_^−^ extracted from CaCO_3_ to form Na_2_CO_3_ (sodium carbonate).

The OA−.20–30 and OA−.30–40 soils; the former was relatively salty, with an electrical conductivity (EC) of 2.84 dS/m, while the latter was not. Both were limestone types. The soil solution was characterized by the presence of Ca^2+^ and Mg^2+^, which were the two dominant cations in the OA−.20–30 soil. Both horizons showed low chemical and biological fertility.

The MN−.20–30 and MN−.30–40 soils were not very salty. The pH was slightly alkaline. The first contained a CaCO_3_ content of over 15%, giving it a carbonated state. The solution had low K^+^ contents and a dominance of Mg^2+^.

- Healthy samples were taken from a healthy palm grove in an infected area

Regarding the TS−.20–30 (1) and TS−.30–40 (1) samples, as well as the TS−.20–30 (2) and TS−.30–40 (2) samples, our results indicated that these horizons were weakly salty, with a relatively alkaline pH. CaCO_3_ content varied from 11.5% to 22.22%. This suggests that high pH levels depend on the concentration of carbonates in the soil solution.

- Healthy samples were taken from a healthy palm grove in a healthy area

Prospecting of the different soil horizons (Or−.20–30 (1), Or−.30–40 (1), Or−.20–30 (2), Or−.30–40 (2), BB−.20–30 (1), BB−.30–40 (1), BB−.20–30 (2), and BB−.30–40 (2)) showed that the alkalinity of the soils increased proportionally with the content of salt and limestone. These soils exhibited high PO_4_^2−^ contents, except for the BB−.30–40 (1) horizon. K^+^ contents were weak. The geochemical facies were dominated by the Ca^2+^ and Mg^2+^.

**FIGURE 2:**
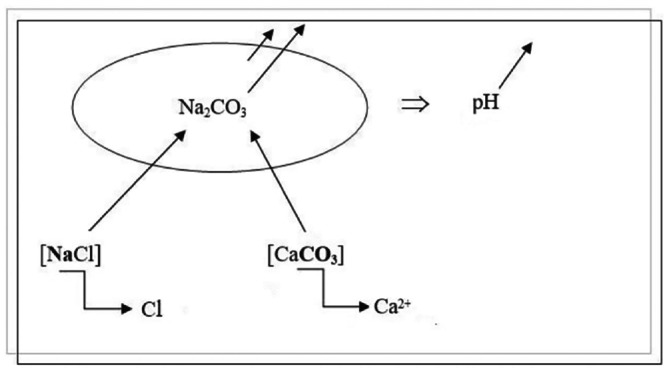
Circumstances of alkalinity of healthy palm grove horizons.

The state of these latter horizons, which are rich in calcium carbonate (limestone) and salt, can be schematized in (Figure 2).

The combination of Na^+^, released by salt, and CO_3_, released by limestone, causes the formation of sodium carbonate (Na_2_CO_3_). This results in an alkaline environment with a pH of around 9.

Table 1 shows the extrema of the different parameters analyzed and their locations according to the aforementioned situations. Each value in Table 1 is the average of three replicates. The results at the 00–20 horizon level did not lead to any definitive conclusions; only samples from horizons 20–30 and 30–40 were considered.

**TABLE 1:** Average of the extrema for the different soil parameters analyzed in the four states considered.

State	Global		State 1		State 2		State 3		State 4	
			Ar.(+) _ Pal.(+) _ Hz.(+)^a^		Ar.(+) _ Pal.(+) _ Hz.(−)^a^		Ar.(+) _ Pal.(−)_Hz.(−)^a^		Ar.(−) _ Pal.(−)_ Hz.(−)^a^	
Parameters	Max (sample)	Min (sample)	Max (sample)	Min (sample)	Max (sample)	Min (sample)	Max (sample)	Min (sample)	Max (sample)	Min (sample)
CE (ds/m)	3.45 BB−.20–30 (2)	0.14 SB−.30–40	1.79 SB+.20–30	0.29 (SB−.20–30 & GH+.20–30)	2.84 OA−.20–30	0.14 SB−.30–40	0.67 TS−.30–40 (2)	0.15 TS−.20–30 (1)	3.45 BB−.20–30 (2)	2.29 OR−.20–30 (2)
pH	8.98 MN+.30–40	7.47 OA−.20–30	8.98 MN+.30–40	7.65 OA+.20–30	8.62 OA−.30–40	7.47 OA−.20–30	8.5 TS−.30–40 (2)	7.84 TS−.30–40 (1)	8.09 OR−.30–40 (2)	7.64 BB−.20–30 (1)
PO_4_^2−^ (ppm)	88.46 BB−.20–30 (2)	3.99 GH+.30–40	30.6 OA+.20–30	3.99 GH+.30–40	71.2 OA−.20–30	7.2 DH−.30–40	18.87 TS−.20–30 (2)	5.02 TS−.30–40 (2)	88.46 BB−.20–30 (2)	7.8 BB−.30–40 (1)
CaCO_3_ (%)	35 OR−.20–30 (1)	7.64 SB−.20–30	26.46 GH+.30–40	9.5 SB+.30–40	22.35 DH−.20–30	7.64 SB−.20–30	22.22 TS−.20–30 (2)	11.5 TS−.30–40 (1)	35 OR−.20–30 (1)	17.5 BB−.30–40 (1)
MO (%)	3.01 BB−.20–30 (2)	0.05 SB−.20–30	1.52 GH+.20–30	0.08 DH+.20–30	1.36 OA−.20–30	0.05 SB−.20–30	1.03 TS−.30–40 (2)	0.33 TS−.30–40 (1)	3.01 BB−.20–30 (2)	0.54 BB−.30–40 (1)
K^+^ (meq/L)	1.01 BB−.20–30 (2)	0.1 SB+.30–40	0.68 OA+.30–40	0.1 SB+.30–40	0.63 MN−.30–40	0.13 SB−.30–40	0.52 TS−.30–40 (2)	0.27 TS−.30–40 (1)	1.01 BB−.20–30 (2)	0.3 BB−.20–30 (1)
Na^+^ (meq/L)	3.88 BB−.30–40 (1)	0.02 DH−.20–30	2.49 MN+.20–30	0.14 SB+.30–40	3.57 MN−.20–30	0.02 DH−.20–30	2.68 TS−.30–40 (2)	0.71 TS−.30–40 (1)	3.88 BB−.30–40 (1)	0.16 OR−.20–30 (2)
Ca^2+^ (meq/L)	18.2 OR−.30–40 (2)	0.8 GH+.20–30	5.2 MN+.30–40	0.8 GH+.20–30	12.1 OA−.20–30	1 SB+.30–40	7 TS−.30–40 (2)	1 TS−.20–30 (1)	18.2 OR−.30–40 (2)	9.6 OR−.20–30 (2)
Mg^2+^ (meq/L)	24.2 BB−.30–40 (2)	0.2 TS−.30–40 (2)	11.2 SB+.30–40	0.6 DH+.30–40	12.7 OA−.20–30	0.4 GH−.20–30	2 TS−.30–40 (1)	0.2 TS−.30–40 (2)	24.2 BB−.30–40 (2)	10.2 OR−.30–40 (2)

^a^ Ar. (+): infected area; Pal. (+): infected palm grove; Hz. (+): infected horizon; Ar. (−): uninfected area; Pal. (−): uninfected palm grove; Hz. (−): uninfected horizon.

Comparing healthy and diseased horizons located in the same infected area revealed no differences. However, comparing horizons in an infected area with those in a healthy area was very conclusive.

**FIGURE 3:**
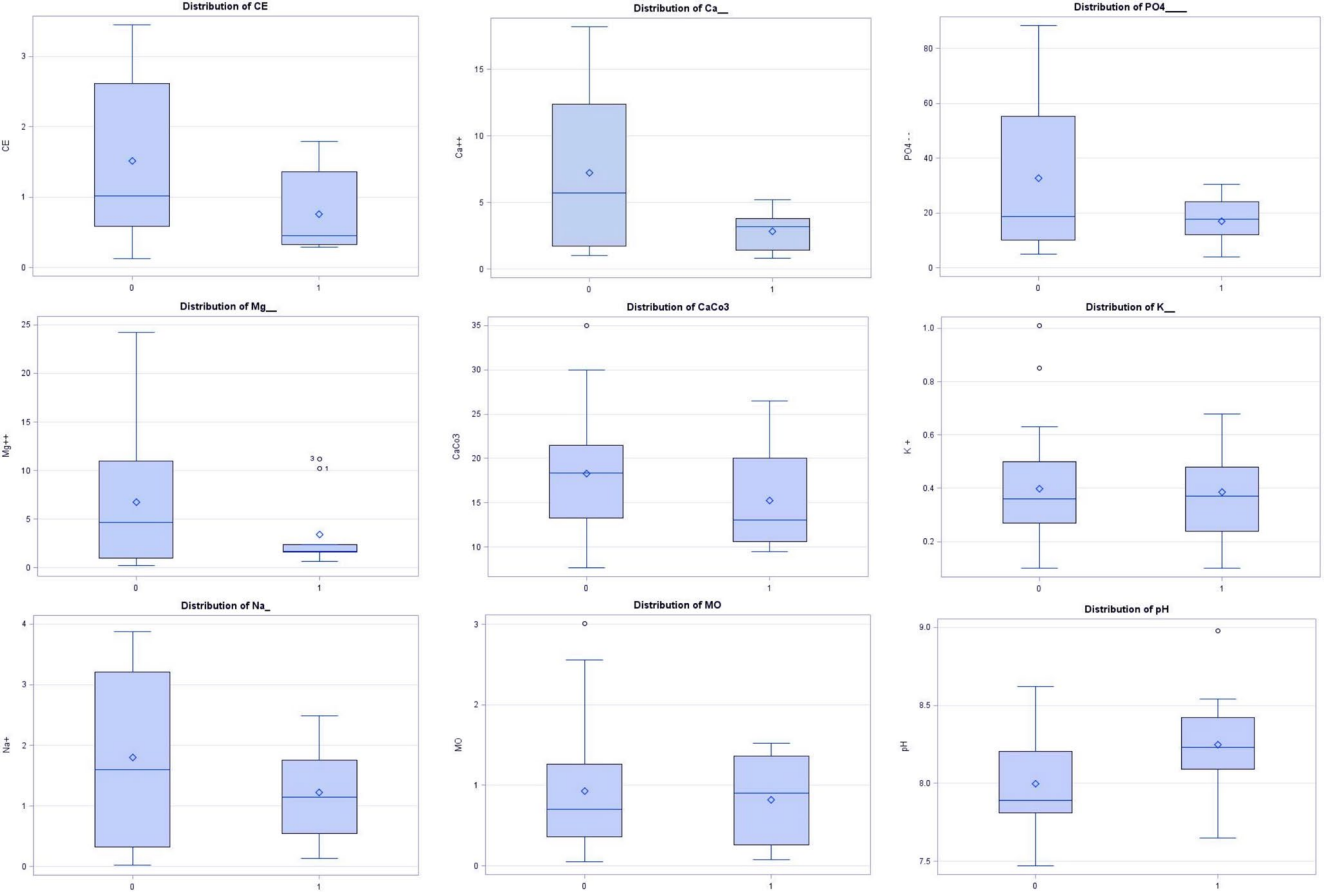
Box plots illustrating the parameters of the chemical analyses of the soil samples studied (0: Healthy, 1: Diseased).

According to the probability levels listed in Table 2, five parameters appear to discriminate between healthy and diseased profiles in infected and uninfected areas, respectively. The differences observed for these parameters are shown in boxplots (Figures 2 and 3). This discrimination suggests that these factors may significantly impact the incidence of the disease. In order of importance of segregation, these factors are CE, Ca^2+^, PO_4_^2−^, Mg^2+^ and CaCO_3_.

**TABLE 2:** Comparison between healthy profiles in an uninfected area and diseased profiles in an infected area; *p*‐values and levels of significations.

Parameters	CE	pH	${\mathrm{PO}}_{4}^{2-}$	${\text{CaCO}}_{3}$	MO	$K^{+}$	${\mathrm{Na}}^{+}$	${\mathrm{Ca}}^{2+}$	${\mathrm{Mg}}^{2+}$
Significance value	< 0.001	0.114	0.004	0.013	0.062	0.195	0.194	< 0.001	0.007
Level of significance	****	No	***	**	No	No	No	****	***

**: significant at 5%; ***: significant at 1%; ****: signifaicant at1‰.

## 4 Discussion

### 4.1 Analysis of pH and EC Values in Relation to Palm Grove Health

The analysis of pH and EC values presented in Table 1 reveals interesting trends related to the health of palm groves. The pH values in diseased areas ranged from 8.98 [MN+.30–40] to 7.65 [OA+.20–30], while healthy areas exhibited values between 8.09 [Or−.30–40 (2)] and 7.64 [BB−.20–30 (1)]. Notably, the pH parameter did not clearly distinguish between healthy and diseased zones, suggesting it is not a reliable indicator of disease presence or absence. However, a significant difference was observed in electrical conductivity. Healthy, noninfected palm groves displayed higher EC values (3.45 [BB−.20–30 (2)] to 2.29 dS/m [Or−.20–30 (2)]), whereas diseased areas had much lower values (1.79 [SB+ 0.20–30] to 0.29 dS/m [SB+ 0.30–40 and GH+ 0.20–30]).

ANOVA confirmed a significant difference in electrical conductivity between healthy and diseased zones, with higher EC values in healthy areas. This suggests that increased electrical conductivity (high salinity) may be unfavorable for the emergence and progression of disease. This aligns with numerous studies showing that high salinity inhibits certain diseases.

The work of several authors supports the idea that elevated EC levels suppress diseases such as *Rhizoctonia* root rot in sugar beets (*Rhizoctonia solani*) (Elmer 1997) and crown and root rot in asparagus (*F. oxysporum* and *F. proliferatum*) (Syn = *F. moniliforme*) (Elmer and LaMondia 1999; Elmer 2003). Banana Fusarium wilt (*F. o. cubense*) also showed higher electrical conductivity in areas where the disease was suppressed (Cogliati et al. 2012). High EC enhances antioxidant activities and increases stress markers such as proline (Dewir and Alsadon 2022), with proline dehydrogenase contributing to pathogen defense (Cecchini et al. 2011). Conversely, low EC negatively impacts plant health and yield (Dominguez et al. 2001; Samarakoon et al. 2006).

Studies on Fusarium wilt in date palms (Amir et al. 1989; Amir and Riba 1990) demonstrated that high salinity (EC = 20 dS/m^−1^) inhibits *Fusarium* populations, particularly pathogenic strains of *F. o. albedinis*. Dossa et al. (2019) showed that sodium chloride stimulates some *Fusarium* species while strongly suppressing others.

However, our results contrast with those of Triky‐Dotan et al. (2005), who observed increased disease severity in tomatoes (*F. o. radicis‐lycopersici*) with rising EC due to saline irrigation. Similarly, studies on *F. o. vasinfectum* (Ragazzi and Vecchio 1992; Turco et al. 2002) and *F. o. cyclaminis* (Elmer 2002) found that high salinity either increased disease severity or had no effect. Chitarra et al. (2013) reported no impact of increased EC on disease in a soilless system.

High NaCl levels, leading to elevated Na^+^ in plant tissues, can disrupt K^+^ and NO_3_^−^ status, thereby affecting cellular functions (Grattan and Grieve 1999; Jēkabsone et al. 2022).

### 4.2 The Role of Macronutrients

Regarding the impact of other parameters represented by the four macronutrients (Ca^2+^, PO_4_^2−^, Mg^2+^, and CaCO_3_), it is important to note that these elements influence both the plant and the soil microbiome. In plants, while disease resistance largely depends on genetics, nutrients contribute to the expression of this resistance or regulate sensitivity to specific pathogens. In the soil, nutrients either stimulate or inhibit inoculum dynamics (Xue et al. 2019).

### 4.3 Calcium Concentration and Disease Resistance

Diseased plants in affected areas showed an average calcium (Ca) concentration of 2.82 meq/L, while healthy plants in the same zones averaged 4.16 meq/L. Healthy zones exhibited even higher Ca levels (10.58 meq/L), suggesting a strong correlation between Ca and disease prevention (Alvarez et al. 1981; Blanc et al. 1983; Jones and Woltz 1970; Sugimoto et al. 2008; Woltz and Engelhard 1973). This aligns with studies on Fusarium wilt in tomatoes (*F. o. lycopersici*), carnations (*F. o. dianthi*), and chrysanthemums (*F. o. chrysanthemi*), where Ca reduced disease severity. Adequate Ca supplementation has even eradicated banana Fusarium wilt in field studies (Alvarez et al. 1981; Woltz and Engelhard 1973). Calcium deficiency weakens plants structurally and functionally, making them more vulnerable to fungal attacks. This manifests as:

– Disrupted plant cell wall formation: leading to lysine degradation, pipecolic acid accumulation, and reduced biomass (Tripathi et al. 2022; Weng et al. 2022).
– Unstable cell membranes: causing leaks of sugars and amino acids, creating an environment favorable for pathogen infection (Marschner 1995).
– Calcium‐unsaturated pectins: pathogens secrete pectic enzymes, such as pectin methylesterase (PME) and polygalacturonase (PG), which degrade cell walls. Ca‐saturated pectins resist enzymatic degradation, whereas substitution with Na^+^ or Mg^2+^ ions increases plant vulnerability (Bateman and Basham 1976; Chimwamurombe 2001; Datnoff et al. 1991; Huber 1980; Nakamura and Iwai 2019; Wei et al. 2022).

High calcium concentrations inhibit pathogens by disrupting their metabolism, reducing sporulation, and limiting fungal enzyme activity (Halsall and Forrester 1977; Posada‐Gómez et al. 2018; Roy et al. 2020; Xu et al. 2013). However, this effect is dose‐dependent: moderate concentrations may stimulate fungal growth, while excess Ca^2+^ becomes cytotoxic to fungi (Roy et al. 2020).

In soil, calcium plays a critical role in microbial antagonism. High free Ca content correlates negatively with disease severity, as seen in its suppressive effect on pea root rot caused by *Aphanomyces euteiches* (Heyman et al. 2007). Additionally, calcium amendments stimulate antagonistic microorganisms, significantly reducing pathologies (Ko and Kao 1989).

In signal transduction, Ca^2+^ acts as a key secondary messenger, activating defense genes in response to biotic (pathogens) or abiotic stress (Cheong et al. 2002, 2003; Navarro et al. 2004; Wang et al. 2019; Xu et al. 2015; Yan et al. 2022). During fungal invasions, cytosolic Ca^2+^ spikes trigger rapid responses (Xu and Heath 1998), and elicitors such as *Phytophthora*'s Pep‐13 induce calcium influx (Blume et al. 2000). These signals are relayed by the Ca^2+^‐calmodulin complex, which plays a central role in microbial signal perception and immune response regulation, linking Ca^2+^ fluctuations to specific defense pathways (Garcia‐Mina 2012; Yuan et al. 2017). Ca‐pectin or Ca‐calmodulin interactions could inspire crop protection strategies.

Furthermore, ethylene‐mediated defense (ET) acts synergistically with calcium pathways: ethylene modulates the expression of defense genes like chitinase and enhances responses to necrotrophic pathogens, often via Ca^2+^‐dependent regulation (e.g., MAPK protein kinase activation) (Broekaert et al. 2006; Pieterse et al. 2012). Experiments with Ca chelators showed that blocking calcium fluxes inhibits these ethylene‐dependent responses (Raz and Fluhr 1992). Thus, Ca^2+^ and ethylene form an integrated signaling network to coordinate plant resistance.

In summary, calcium plays a key, multifunctional role in protecting plants against infections and enhancing pathogen resistance. It acts indirectly by strengthening host defense mechanisms—serving as a structural stabilizer and central coordinator of immune signaling pathways—and directly by inhibiting pathogens and regulating beneficial microbial interactions in the soil. The presence of other anions and cations in the nutrient solution does not modify the main effect of Ca on the suppression of wilting syndrome (Spiegel et al. 1987).

With regard to PO_4_^2−^, our results corroborate the conclusions of several studies demonstrating the protective effects of phosphorus. This is illustrated by studies in which potassium phosphate (KH_2_PO_4_) reduced powdery mildew in barley by approximately 70% (Mitchell and Walters 2004) and in which triple superphosphate significantly reduced the incidence of potato scab, although through an indirect mechanism, as the pathogen itself is not inhibited by phosphorus (Davis et al. 1976). In addition, phosphorus fertilization has almost eliminated *Pythium*‐induced root rot in wheat (Huber 1980), and combinations of lime, nitric nitrogen, and phosphorus have been shown to be effective against Fusarium head blight (Ojha and Jha 2021). However, this role is nuanced, as there are exceptions, such as in the case of the fungus responsible for rice blast (*Magnaporthe oryzae*) under conditions of excess phosphate (Campos‐Soriano et al. 2020) or the aggravation of wilt severity in cotton and melon (Jones et al. 1989), highlighting the contextual nature of these interactions.

The protective effect of phosphate is orchestrated through a dual mechanism. Primarily, it empowers the plant's beneficial microbiome. Thus, phosphate creates a synergistic environment where it strengthens both the plant's defenses and the antagonistic action of the beneficial microbiome. This is achieved by supporting rhizobacteria, such as *Pseudomonas*, which are pivotal in initiating induced systemic resistance (ISR). This state of heightened alert, mediated by signaling hormones like jasmonic acid and ethylene, allows the plant to mount a more rapid and effective counterattack against pathogen invasion. Crucially, phosphate availability amplifies these defense signals, making the plant more receptive to threats (Berendsen et al. 2012; Siddiqui et al. 2015; Schlatter et al. 2017).

Simultaneously, phosphate provides the essential energy and metabolic precursors for these same beneficial bacteria to produce siderophores. These high‐affinity iron‐chelating molecules sequester ferric iron (Fe^3+^), instigating a fierce nutritional competition that starves pathogenic fungi of this vital resource, thereby curbing their growth and infectivity (Mitra et al. 2020; Cao et al. 2024). The direct effects of phosphorus on pathogens are equally complex; foliar applications of KH_2_PO_4_ solutions have shown systemic protection against various diseases, inhibiting pathogens like *Puccinia sorghi* (corn rust) and *Podosphaera leucotricha* (apple powdery mildew) (Reuveni and Reuveni 1998; Reuveni, Oppenheim, and Reuveni 1998; Reuveni, Dor, and Reuveni 1998). Furthermore, specific concentrations can reduce sporulation in fungi like *Claviceps purpurea* by 50% (Kybal et al. 1968) and inhibit key fungal enzymes such as phosphatases (Bernard et al. 2002; Tibbett et al. 1998; Yoshida et al. 1989), though sometimes creating trade‐offs between pathogen growth and reproduction, as seen in *Helminthosporium solani* (Garraway and Evans 1984).

Finally, the impact of phosphorus is profoundly mediated by the soil ecosystem. While high phosphorus levels are known to reduce root colonization by arbuscular mycorrhizal fungi (AMF) (Garbaye 2013), a phenomenon well‐documented across multiple studies (Abbott and Robson 1984; Amijee et al. 1989; Koide and Li 1990), other microbial allies like phosphate‐solubilizing bacteria (PSB) can mitigate these effects by improving phosphorus availability in the rhizosphere (Korir et al. 2017; Yu et al. 2012). This illustrates that the ultimate effect of phosphate on plant health is a multifaceted interplay between the specific pathosystem, application method, and the structure and function of the soil microbial community. In summary, phosphorus is not merely a fertilizer but a key orchestrator in the complex web of plant defense, microbial ecology, and pathogen dynamics.

Exchangeable magnesium has been identified as a discriminant factor in soils from healthy versus diseased areas. This finding aligns with studies on *F. o. cubense*, the causal agent of banana Fusarium wilt (Alvarez et al. 1981; Júnior et al. 2000), which have highlighted its pronounced influence in surface soils. Similarly, healthy grapefruit plants (*Citrus paradisi*) exhibited higher boron (B) and magnesium (Mg) concentrations compared to those affected by Huanglongbing (Ferrarezi et al. 2022).

The suppressive or mitigating effects of magnesium on diseases are well documented across various crops, including oil palms infected with *Ganoderma boninense* (Akbar et al. 1971) and rice affected by brown spot disease caused by *Bipolaris oryzae* (Moreira et al. 2015).

However, the exact mechanisms of action remain controversial. While Jones and Huber (2007) suggest that the role of magnesium is nonspecific and related to general nutritional and physiological functions of plants, Huber and Jones (2013) note that deficiencies lead to an accumulation of sucrose and amino acids in the leaves, creating favorable conditions for pathogens.

Magnesium contributes to disease resistance by modulating defense enzyme activity (Moreira et al. 2013; Ahmed et al. 2023) and ensuring sufficient energy production to counteract pathogenic metabolites (Tripathi et al. 2022). Its availability in soils is influenced by factors such as soil type and competing cations, including calcium in limestone soils, hydrogen and ammonium in acidic soils, and aluminum, manganese, or sodium in saline or potassium‐rich soils (Garcia et al. 1978; Mengel and Kirkby 2001; Shaul 2002; Cakmak and Kirkby 2008; Zambolim et al. 2019). Additionally, low cation exchange capacity (CEC) in sandy soils exacerbates magnesium deficiency (Cakmak and Kirkby 2008). The element's effects are concentration‐dependent, with higher levels reducing mycotoxin accumulation in *F. graminearum* without hindering mycelial growth (Pinson‐Gadais et al. 2008) and impacting pathogen invasion pathways (Tripathi et al. 2022). Magnesium also plays a role in zoospore formation in *Phytophthora*, though its presence can affect infectivity (Halsall and Forrester 1977).

Recent studies highlight the potential of magnesium oxide nanoparticles (MgO NPs) in disease control, showing antifungal activity against *Fusarium oxysporum* f. sp. *lycopersici* and inhibition of *Bacillus* biofilm formation (Abdel‐Aziz et al. 2020; Abdul‐Karim 2021; Parizi et al. 2014; Oknin et al. 2015). These effects are dose‐dependent, with higher concentrations yielding greater control (Smith et al. 2003). Despite these benefits, magnesium's efficacy varies depending on pathogen species, environmental conditions, and interactions with other factors, underscoring the need for further research to optimize its use in agriculture.

Calcium carbonate (CaCO_3_) has been demonstrated to have a significant impact on plant pathogens, particularly fungi. Its application to soil reduced the germination of chlamydospores of *Fusarium oxysporum* f. sp. *cubense* and decreased the severity of banana Fusarium wilt by one‐third to one‐half in both suppressive and disease‐prone soils (Peng et al. 1999). Similarly, the addition of calcium carbonate to cabbage seed treatment significantly increased resistance to black rot disease (Hsiao et al. 2023). Studies on Fusarium rot caused by *F. o. radicis‐lycopersici* on tomato showed that crown rot was more severe at the lowest CaCO_3_ level (Woltz et al. 1992). This corroborates the observation of a significant difference in calcium carbonate levels between areas infected by Fusarium wilt of the date palm and healthy areas, suggesting that a high concentration of CaCO_3_ creates an unfavorable environment for the pathogen and is a limiting factor in inoculum dynamics (Figure 3).

The inhibitory effect of calcium carbonate on fungal development is supported by multiple studies. An evaluation of six *Fusarium* spp., including *F. oxysporum*, revealed a significant reduction in conidia production, germination, and mycelium dry weight in the presence of precipitated calcium carbonate in the growth medium (Chittem et al. 2016). Furthermore, most calcium salts tested, including CaCO_3_, were found to inhibit the development of apple rot in vivo and in vitro (Attrassi et al. 2008). CaCO_3_ has also been reported to be very effective in reducing sporulation time and improving heat resistance (Mah et al. 2008). However, the effect can be concentration‐dependent and vary by compound. Analysis of the effect of inorganic carbon compounds on *F. o. lycopersici* showed that unlike sodium carbonate (Na_2_CO_3_), calcium carbonate did not show a significant effect, though high concentrations did have a significant impact on pathogen development (Akram et al. 2023). This inhibitory effect extends beyond pathogens, as a high concentration of CaCO_3_ can also negatively influence the level of mycorrhizal colonization (Destinoble 2017). The lowest sporulation of *Bacillus subtilis* and *B. coagulans* was also observed on media enriched with the highest level of calcium and low levels of manganese (Sinnelä et al. 2019).

A primary mechanism for this inhibitory effect is the ability of CaCO_3_ to alter the pH and nutrient availability in the growing medium. *Fusarium* species primarily require an acidic pH for optimal growth and sporulation. An acidic pH is most suitable for *F. oxysporum* and *F. solani*, and it has been demonstrated that *Gibberella fujikuroi* and *F. oxysporum* grow and sporulate well at a pH of 5–5.5 (Ajmal et al. 2023). A similar result was noted for *F. o. lini*, for which the best pH for growth and sporulation was 5.5–7 (Pal et al. 2019). Calcium carbonate is a known source of alkalinity (Müller et al. 2016; Gaines et al. 2023); its presence can exert a buffering action that raises the pH (Alvarez et al. 1981), thereby creating conditions unfavorable for acid‐preferring fungi.

Finally, the natural presence and formation of calcium carbonate in soil is itself influenced by biological activity. The impact of the microbiome in CaCO_3_ sedimentation or mineralization has been the subject of research in several works (Zheng 2021; Rui and Qian 2022).

**FIGURE 4:**
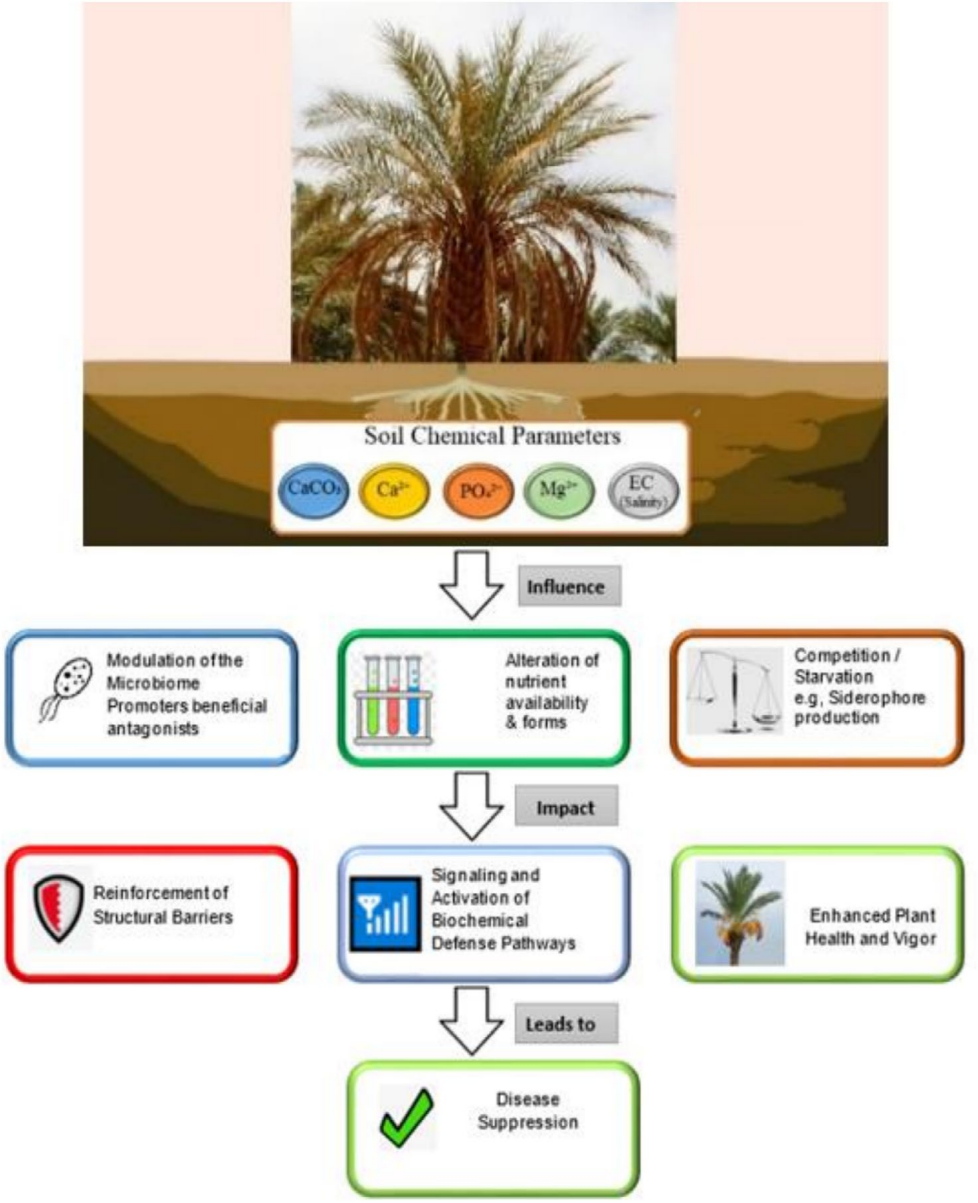
Conceptual diagram summarizing the hypothetical mechanism of disease suppression via studied soil chemical parameters.

A summary of the hypothetical mechanism of disease suppression via the soil chemical parameters studied is shown in Figure 4.

## 5 Conclusion

The comparative analysis of soils affected by Fusarium wilt in date palms versus unaffected soils highlights the significant role of soil chemical properties—such as electrical conductivity, soluble cations, calcium carbonate, and assimilable phosphorus—in influencing disease severity and the dynamics of its causal agent. These nutrients can impact the disease either indirectly, by enhancing the host plant's resistance, or directly, by suppressing the pathogen's growth and sporulation.

However, the effects of individual nutrients are complex and variable, often depending on fungal species, plant host, environmental conditions, and the soil's broader chemical and mineralogical profile. Conflicting results reported in the literature are likely due to these varying experimental conditions and host‐pathogen‐environment interactions. Thus, the role of each nutrient must be interpreted within its specific agro‐ecological context. A single nutrient may reduce disease severity in one scenario while exacerbating it in another.

Moreover, both the form and concentration of nutrients are relevant to the distribution and survival of the pathogen inoculum, although no clear nutrient gradient could be established. While plant nutrition remains a promising avenue for disease management, current data are fragmented, and further in‐depth research is needed to clarify these complex relationships.

Future studies should broaden the scope to include more nutrients, locations, and soil horizons. Critical research questions include whether Fusarium wilt progression is driven primarily by soil properties, pathogen variability, or host plant distribution, and how the plant microbiome may influence disease spread and intensity. Soil microbiological analyses could be key to uncovering potential links between microbial communities and pathogen dynamics. These avenues will be explored in future research efforts.

## Conflicts of Interest

The authors declare no conflicts of interest.

## Data Availability

The data supporting the findings of this study are openly available in Djekiref et al. (2025) at https://doi.org/10.5281/zenodo.16980545.
